# Abortion hotlines around the world: a mixed-methods systematic and descriptive review

**DOI:** 10.1080/26410397.2021.1907027

**Published:** 2021-04-29

**Authors:** Roopan K. Gill, Amanda Cleeve, Antonella F. Lavelanet

**Affiliations:** a Consultant, UNDP-UNFPA-UNICEF-WHO-World Bank Special Programme of Research, Development and Research Training in Human Reproduction (HRP), Department of Sexual and Reproductive Health and Research, World Health Organization, Geneva, Switzerland; Clinical Assistant Professor, Department of Obstetrics and Gynecology, University of Toronto, Toronto, Canada; b Consultant, UNDP-UNFPA-UNICEF-WHO-World Bank Special Programme of Research, Development and Research Training in Human Reproduction (HRP), Department of Sexual and Reproductive Health and Research, World Health Organization, Geneva, Switzerland; Department of Women’s and Children’s Health, Karolinska Institutet, Solna, Sweden; South General Hospital, Stockholm, Sweden; c Medical Officer, Department of Sexual and Reproductive Health and Research and UNDP-UNFPA-UNICEF-WHO-World Bank Special Programme of Research, Development and Research Training in Human Reproduction (HRP), World Health Organization, Geneva, Switzerland

**Keywords:** abortion, hotlines, harm reduction, legal restrictions, systematic review

## Abstract

Barriers to access abortion services globally have led to the development of alternative methods to assist and support women who seek an abortion. One such method is the use of hotlines, currently utilised globally for abortion care. This review aimed to understand (1) how abortion hotlines facilitate access to abortion; and (2) how women and stakeholders describe the impact of hotlines on abortion access. Published quantitative and qualitative studies and grey literature were systematically reviewed alongside an identification and description of abortion hotlines in the public domain. Our findings highlight that the existence of abortion hotlines is highly context-dependent. They may exist either as an independent community-based model of care, or as part of formal care pathways within the health system. Hotlines operating in contexts with legal restrictions seem to be broader in scope and will use innovative approaches to adapt to their setting and reach hard-to-reach populations. All the abortion hotlines that provided information on a data extraction form used evidence-based guidelines but women seeking medical abortion still struggle to access quality medications. There is limited data in general on abortion hotlines, especially on the user and provider experience. Abortion hotlines have the potential to facilitate access to safe abortion care through evidence-based information and to decrease maternal mortality and morbidity from unsafe abortions for women and girls globally.

## Résumé

Les obstacles à l’accès aux services d’avortement dans le monde ont incité à mettre au point des méthodes de rechange pour aider et soutenir les femmes qui veulent avorter. Une de ces méthodes est le recours aux services d’information téléphonique, actuellement utilisés dans le monde pour l’avortement. Notre étude visait à comprendre: (1) comment ces numéros d’appel facilitent l’accès à l’avortement; et (2) comment les femmes et les parties concernées décrivent l’impact de ces lignes sur l’accès à l’avortement. Les études quantitatives et qualitatives publiées et la littérature grise ont fait l’objet d’un examen systématique, parallèlement à l’identification et la description de services d’information téléphonique dans le domaine public. D’après nos conclusions, l’existence de lignes d’information dépend étroitement du contexte. Ces services peuvent être proposés soit dans un modèle de soins communautaire indépendant, soit dans des filières de soins formelles dans le cadre du système de santé. Les lignes d’information opérant dans des contextes avec des restrictions légales semblent avoir une portée plus large et utilisent des approches novatrices pour s’adapter à leur environnement et desservir les populations difficiles à atteindre. Toutes les lignes téléphoniques sur l’avortement qui offraient des informations sous la forme d’extraction de données utilisaient des directives à base factuelle, mais les femmes qui souhaitent un avortement médicamenteux ont encore du mal à avoir accès à des médicaments de qualité. On dispose en général de données limitées sur les lignes téléphoniques relatives à l’avortement, mais tout particulièrement sur l’expérience des usagers et des prestataires. Les services d’information téléphonique sur l’avortement ont le potentiel de faciliter l’accès à un avortement sûr par des renseignements concrets, tout en diminuant la mortalité et la morbidité maternelles dues aux avortements à risque que subissent les femmes et les jeunes filles dans le monde.

## Resumen

En respuesta a las barreras para acceder a los servicios de aborto a nivel mundial, se han creado métodos alternativos para ayudar y apoyar a las mujeres que buscan un aborto. Uno de esos métodos es el uso de líneas de atención telefónica, actualmente utilizadas mundialmente para los servicios de aborto. El objetivo de esta revisión era entender (1) cómo las líneas de atención telefónica sobre aborto facilitan el acceso a los servicios de aborto; y (2) cómo las mujeres y partes interesadas describen el impacto de las líneas de atención telefónica en el acceso a los servicios de aborto. Se revisaron sistemáticamente estudios cuantitativos y cualitativos publicados y literatura gris junto con la identificación y descripción de las líneas de atención telefónica sobre aborto en el dominio público. Nuestros hallazgos destacan que la existencia de líneas de atención telefónica sobre aborto depende mucho del contexto. Pueden existir como modelo de atención comunitario independiente, o como parte de las rutas de atención formal dentro del sistema de salud. Las líneas de atención telefónica que funcionan en contextos con restricciones legislativas parecen tener un alcance más amplio y utilizan enfoques innovadores para adaptarse a su entorno y llegar a las poblaciones difíciles de alcanzar. Todas las líneas de atención telefónica sobre aborto que proporcionaron información en un formulario de extracción de datos utilizaron guías basadas en evidencia, pero las mujeres que buscan un aborto con medicamentos aún tienen dificultades para acceder a medicamentos de calidad. En general, hay datos limitados sobre las líneas de atención telefónica sobre aborto, especialmente sobre la experiencia de usuarias y prestadores de servicios. Las líneas de atención telefónica sobre aborto tienen el potencial de facilitar el acceso a servicios de aborto seguro por medio de información basada en evidencia y disminuir la morbimortalidad materna de mujeres y niñas a causa de abortos inseguros a nivel mundial.

## Introduction

Approximately 73 million abortions occurred each year between 2015 and 2019,^1^ and yet access to comprehensive abortion care is compromised. Several factors, including poor availability of good quality services, high cost, stigma, and conscientious objection among healthcare providers, result in unsafe abortion being one of the leading causes of maternal mortality and morbidity worldwide.^2,3^ Restrictive laws, regulatory barriers and unnecessary requirements designed to delay and restrict access compound the issue, with greater impacts on those who are already marginalised.^2–4^ Access to safe and effective abortion is an essential component of sexual and reproductive health and all individuals are entitled to the basic human right to make decisions about when and if they desire to be pregnant.

To help individuals mitigate some of these challenges in accessing abortion, community-based organisations, advocates, healthcare providers, and feminist groups around the world have developed innovative models to improve access to safe abortion care, providing evidence-based information to facilitate self-managed abortion care. Given the barriers that individuals face in accessing abortion, these models often work in parallel to, or are integrated within, the formal health system.^5,6^ For example, harm reduction programmes (some of which exist in clinics) are available in several countries,^7–9^ including those programmes that facilitate access to information related to self-management of medical abortion, expand patient knowledge and raise awareness related to the law. These and Web-based platforms are also used in countries like the United States (USA) and Canada, and in many parts of Europe, to facilitate service delivery, sometimes in combination with task sharing (defined as the expansion of health providers who can appropriately deliver health services^10^) for direct provision of abortion medications.^4,6,11–13^ Some organisations even provide in-person accompaniment, where evidence-based counselling and support through the medical abortion process is given to individuals throughout their abortion experience.^9^

Hotlines are a tool used historically to facilitate access to sexual and reproductive health care. HIV/AIDS hotlines were initially set up to provide a confidential avenue to discuss sensitive issues while also addressing geographic barriers to accessing timely care.^17–19^ Hotlines are fairly simple and accessible tools that can be utilised by anyone who has a landline, cellphone or access to a digital device where contact can occur by voice, text message or chat, in an anonymised fashion. This is particularly useful for stigmatised sexual and reproductive health issues such as HIV/AIDS, abortion and gender-based violence.^17,18^

There is a growing body of literature on abortion service delivery via telemedicine, harm reduction and accompaniment models (provision of counselling and support) of care, but questions still remain about abortion hotlines.^4,12,15,16^ Abortion hotlines are defined as “an information service whose purpose is to promote access to safe abortions, offering women information by telephone about how to terminate a pregnancy using medications based on evidence-based protocols”.^14^ Some studies have demonstrated a positive impact,^14^ but greater knowledge is needed about how hotlines operate and facilitate access to abortion, and what barriers and/or facilitators may exist related to the co-existence of hotlines with formal health systems. For the most part, hotlines are run by grassroots feminist organisations to fill a gap in the health system and ensure that women have access to a quality abortion experience, from managing the experience to guidance for after-care.^4,12,15,16^ No systematic review synthesising their findings exists and questions remain on how abortion hotlines operate and facilitate access to abortion, how context conditions their existence, and how hotlines are perceived by users and providers.

To address these knowledge gaps, we aimed to identify possible areas for future research, by understanding how abortion hotlines facilitate access to abortion and how women and stakeholders describe the impact of hotlines on abortion access. This work was commissioned as part of the World Health Organization (WHO) update to the *Safe abortion: technical and policy guidance for health systems* guideline.^20^

## Methods

We conducted this study using two approaches: (1) we systematically reviewed published quantitative and qualitative studies and grey literature, and (2) we identified and described abortion hotlines in the public domain, using evidence synthesised from a data extraction form. This included hotlines which can be searched online or in published data. By reviewing the published and grey literature, we specifically sought to understand: how abortion hotlines facilitate access to abortion, including the types of services provided, types of services sought and standard operating features; and how women and stakeholders describe the impact of hotlines on abortion access. We were particularly interested in how women used and perceived the services as well as hotline staff’s perspectives on providing hotline services. Using the data extraction form for evidence synthesis, we collected information from hotlines operating in the public domain to assess: the number of hotlines that are publicly accessible; what information the hotlines provide; what types of providers staff the hotlines and what training they receive; how evidence-based protocols are used; how hotlines are used to facilitate access; and how those managing hotlines view the role of hotlines in the larger context of abortion provision. For the purposes of this study, we distinguished between hotlines providing services as a component of the formal care pathway and those that are community-based, operating outside the formal health system.

### Literature review

#### Search strategy

We searched published literature in PubMed, CINAHL, Embase, PsychINFO, Global Index Medicus, Popline and EBSCO, using controlled vocabulary and free-text terms and combining MeSH terms. The authors drafted the original search strategy; a specialist librarian at WHO refined the strategy. We conducted the original search on 3 April 2019 and updated the search on 30 April 2020. We used the search engines Google Scholar, Google, Yahoo, Bing and OpenGrey for grey literature. The search strategy is available (Supplemental data 1).

#### Study selection

We included qualitative and quantitative studies without restrictions on language, geography or date published. Two independent reviewers (RG, AC) screened each title and abstract for inclusion using standardised inclusion criteria: (a) hotlines that exist within the public domain, which include those that can be searched via internet search engines or through international or national organisations’ websites; (b) sexual health hotlines that include abortion; (c) abortion-specific hotlines; and (d) quantitative, qualitative and mixed-methods studies. The full text was obtained if both reviewers judged a citation to be potentially eligible. Any discrepancies during screening were resolved by discussion between the two reviewers and if needed, with a third reviewer (AL), until consensus was reached.

#### Data extraction

We adapted a standardised data extraction form, which included the following domains: study identification, methods and population. We extracted data related to the pre-specified descriptive outcomes from the quantitative studies. The outcomes of interest were grouped as: services provided by the hotline; training of the hotline staff; educational background of hotline staff; use of evidence-based guidelines; and types of services sought by callers. From the qualitative studies, we extracted data from the findings and discussion sections. We were interested in how women used and perceived the services as well as hotline staff’s perspectives on providing hotline services.

#### Synthesis

We used a thematic analysis approach to synthesise the qualitative data using Atlas Ti.^21^ One reviewer (RG) conducted open coding to develop an initial set of codes, which were then reviewed by AC and AL. Once the codes were refined, we collated the codes into sub-themes and merged them into core themes. We used an iterative process to develop the sub- and core themes. We did not develop high level analytic themes because of limitations related to the quality and amount of data available.

#### Quality assessment

We assessed the quality of the qualitative studies using an adaptation of the Critical Appraisal Skills Programme (CASP) quality-assessment tool (http://www.casp-uk.net). Assessment included the following domains: aims, methodology, design, recruitment, data collection, data analysis, reflexivity, ethical considerations, findings and research contribution. The modified CASP is used to appraise qualitative studies based on three broad areas: (1) Are the results of the study valid? (Section A) (2) What are the results? (Section B) 3. Will the results help locally? (Section C) We have attached a table as a supplemental document that provides the detailed assessment. The overall utility of CASP is to appraise the validity of the qualitative studies based on the above domains. (Supplemental data 2) Two reviewers (RG, AC) independently evaluated the quality assessment by answering “yes”, “no”, “can’t tell” for 10 questions across the three broad areas. Overall quality assessment was scored using “high”, “medium” and “low” based on discussion between the reviewers on their independent answers; discrepancies were discussed with the third reviewer (AL). We attempted to critically appraise the study designs of included quantitative studies using the Newcastle Ottawa Scale for observational studies. However, our outcomes of interest pertained to the description of the hotlines and not to the outcomes reported by the authors. In addition, as the study designs did not lend to adaptation of the Newcastle-Ottawa Scale it was not used.

### Review of abortion hotlines

#### Data extraction form

We adopted a similar approach that would be used for data extraction for published literature and developed a data extraction form in English for evidence synthesis. These forms were created and revised based on technical consultations with individuals with expertise on the implementation of hotlines and included information that would be expected in internal policies and procedures. We translated the final form into Spanish and French, as these were the predominant languages utilised based on a preliminary assessment (Supplemental data 3); the form included check boxes and free text responses. The form focused on types of services provided, best practice manuals and the use of evidence-based guidelines, hotline staff and training, and strategic organisational activities to engage marginalised and vulnerable populations.

#### Dissemination

We sent the data extraction form by email through existing reproductive health networks which, at that point in time, included 23 hotlines representing Latin America, Africa, South Asia and Europe. In addition, authors searched the internet for grey literature related to abortion hotlines available in the public domain (those that are searchable from the internet).

#### Synthesis

We synthesised numerical data from the extraction form in tabular format; for all other information we used narrative syntheses.

#### Ethics

Ethics approval was not sought. The review collected literature including unpublished policies, procedures, and guidelines, and assessed existing internal documentation using a data extraction form. Information was only collected from hotlines with publicly available contact information. Information yielded by key informants related to the organisations’ professional functions and practices. Key informants were informed that their information would be used in the review and that by incorporating and sharing their organisations’ information, implied consent was provided.

## Results

### Literature review

**Figure 1.**
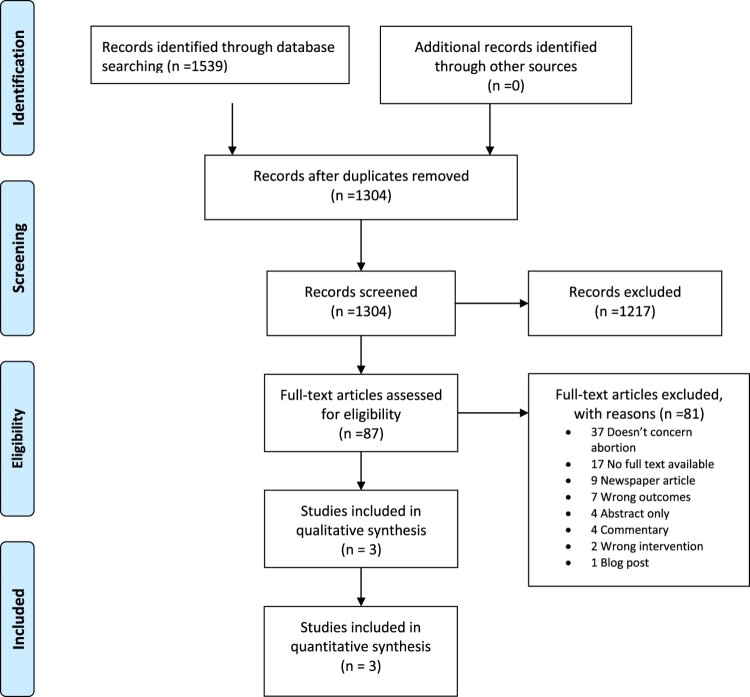
PRISMA flow diagram

The initial search yielded 1539 articles. After duplicates were removed, 1304 remained of which 87 full-text articles were assessed for eligibility. In the end, a total of six articles were included after accounting for exclusions (Figure 1). Of these studies, three were retrospective analyses of hotline user data^11,22,23^ and three were qualitative interview studies.^14,24,25^ The six studies^11,14,22–25^ were conducted in eight countries: Argentina, Canada, Chile, Ecuador, Indonesia, Peru, the USA and Venezuela. Participants in these studies included healthcare providers, abortion seekers, partners, friends and relatives, feminist activists and counsellors. Four studies described community-based abortion hotlines (i.e. hotlines facilitating abortions taking place outside the formal health system) in Latin America and Indonesia.^11,14,22,25^ Two studies described hotlines that were integrated within the formal health system operating in Canada^23^ and the US.^24^ Characteristics of the six included studies are presented in Table 1. Quality assessments of the three included qualitative studies are presented in Table 1.

**Table 1.** Characteristics and quality assessment of included studies

Author, Year	Methods	Participants	Setting: Hotline	Category	Quality assessment of qualitative studies
Casas, 2014^25^	Semi-structured interviews; statistics on hospitalisations and maternal deaths, prosecutions, court cases and cases of people in jail due to abortion	Abortion healthcare providers (*n* = 8); abortion hotline staff; women with experience of illegal abortions (*n* = 41), and their partners, friends and relatives (*n* = 12)	Chile: Linea Aborto Libre	Community-based	Low
Drovetta, 2015^14^	Participatory observation of hotline activities; in-depth interviews; review of materials provided by the hotlines (documents and reports, social media posts, details of public demonstrations and statements)	Feminist activists (*n* = 10); Women who used information provided by these hotlines to induce their own abortions (*n* = 14); Hotline staff (*n* = 5)	Argentina: Abortion Hotline: More information, less risks Chile: Abortion Hotline Chile & Free Abortion Hotline Ecuador: Women’s Health Collective Peru: Dependable Information Abortion Hotline, Venezuela: Abortion Hotline, Dependable Information	Community-based	Low
Gerdts, 2016^11^	Retrospective descriptive analysis of data collected by hotline (volume of calls received, sociodemographic characteristics, abortion-related characteristics)	Callers to an abortion hotline (*n* = 1829)	Indonesia: Samsara	Community-based	N/A
Gerdts, 2018^22^	Retrospective analysis of anonymised electronic client records	Callers to an abortion hotline (*n* = 96)	Indonesia: Not specified	Community-based	N/A
Kimport, 2012^24^	Focus group discussions and in-depth interviews	Talkline counsellors and staff from four abortion support talklines (*n* = 20)	United States of America: Talklines A – D	Component of formal care pathway	Medium
Norman, 2014^23^	Retrospective analysis of programme data from the implementation of an abortion helpline	Callers to a “pregnancy options service” (POS) helpline (*n* = not specified) from 1998 to 2008.	British Columbia, Canada: POS Helpline	Component of formal care pathway	N/A

Of the six studies, four specified the types of services provided by the hotlines.^11,22–24^ The most common services included: pregnancy options counselling, pre-abortion counselling, information about how to take abortion medications and post-abortion counselling.^11,22–24^ In four of the six studies, WHO guidelines were used as a source of information for hotline users.^11,14,22,25^ Four studies highlighted that the most frequent concern for callers was related to medical abortion, including how to access abortion medications and how to use them safely.^11,14,22,25^ Referral to in-person care was either not specified or unclear from all studies aside from one.^23^ Of all the hotlines, only Talkline D identified in Kimport et al’s study included paid counsellors, whereas all others were run by volunteers.^24^ Finally, only one study specifically addressed safety and effectiveness of abortion when facilitated by a hotline.^22^ Details of the outcomes of interest are included in Table 2.

**Table 2.** Outcomes of interest

	Casas 2014	Drovetta 2015	Gerdts 2016	Gerdts 2018	Kimport 2012	Norman 2015
**Service provided by the hotline**						
Pregnancy options counselling	–	–	√		√	√
Pre-abortion counselling	–	–	√	√	√	√
Referral to in-person care	–	–	–	–	–	√
Information about clinics that provide in-person care	–	–	–	–	–	√
Information on where medications can be acquired	–	–	–	√	√	–
Information about how to take medication	–	–	√	√	√	–
Sending abortion medications	–	–	–	X	–	–
Accompaniment at home or in-clinic	–	–	X	√	–	–
Post-abortion counselling and follow-up	–	–	√	√	√	–
Other				Inform about how to communicate with medical personnel	Post abortion emotional support	
**Training of the hotline staff**						
Specified, Training provided	–	–	–	Training in medication abortion protocols and abortion counselling	Talkline A and B – volunteers trained in peer-based counselling, Talkline C and D clergy trained in abortion-related information	–
Educational background of hotline staff						
Education	–	–	–	Not clinically trained	–	–
**Uses evidence-based guidelines**						
Guidance	WHO guidance^20,26^	WHO^20,26^ and national guidance	WHO guidance^20,26^	WHO guidance^20,26^ Gynuity clinical guidelines	–	X
**Types of services sought by hotline callers**						
Pregnancy options counselling	–	–	√	–	√	√
Pre-abortion counselling	√	√	√	√	√	√
Referral to in-person care	–	–	√	–	–	√
Information about clinics that provide in-person care	√	√	–	–	–	–
Surgical abortion	–	–	√	–	–	–
Medical abortion and medications	√	√	√	√	√	–
Post-abortion counselling and follow up	–	–	√	√	√	–

Note: √** = **yes; X** **= No; **– **= Not specified or unclear.

Three studies^14,24,25^ used qualitative methodology and described the historical, legal and sociocultural contexts in which abortion hotlines function, along with their role as political or apolitical tools. Two of these studies discussed hotlines in Latin America, where abortion is legally restricted, and the other study focused on hotlines in the US.^14,24,25^ The analysis of these studies generated three core themes, which are presented below. These themes highlight the role that hotlines play in diverse contexts, what types of information they provide, how the work of hotlines is connected to grassroots feminist movements, sustainability challenges, and the potential link between formal health systems and informal grassroots community organisers.

#### Theme 1: context conditions the existence of hotlines

We found that hotlines existed and were implemented because of perceived unmet reproductive health needs. Why hotlines were implemented, what services they provided and how, and the conditions they operated under, were highly contextual.^14,24,25^

Hotlines in legally restrictive settings were explicitly implemented by women’s collectives to address legal and social barriers to abortion care, with the aim of generating momentum in shedding light on the clandestine nature of abortion care in these settings. The hotlines were an attempt to decrease unsafe abortion and reduce maternal mortality and morbidity, as well as a tool for advocacy to demonstrate the role of hotlines in providing quality care in the absence of formal care provision.^14,25^ According to Casas and Vivaldi, “feminist groups have been crucial not just in putting abortion on the agenda, but also in generating support networks to ensure abortion takes place under less dangerous conditions”.^25^ The hotlines operated in parallel to the formal health system and provided an avenue to facilitate safer self-management. While they did not provide abortion pills directly, these hotlines enabled abortion seekers to safely use abortion pills. Despite providing services remotely (online or phone-based), hotlines in settings where abortion is legally restricted and/or stigmatised were able to offer a wide variety of services, including: eligibility screening, providing information about the dose and route of administration for misoprostol, what symptoms to expect, level of efficacy, and possible side effects.^14,25^ In addition, some hotlines offered accompaniment services and prepared women for their communication with healthcare providers, should they experience complications that required medical attention. This was done in order to protect women from any legal repercussions if the abortion was disclosed.^14^ Given that they function within legally restrictive settings, hotline managers and counsellors themselves were more often at greater risk of criminal investigations and prosecution due to the visibility gained from their advocacy work.^14,25^ For instance, one hotline was blocked by a court order and in another three counsellors were prosecuted.^14,25^

In contrast, hotlines in less restrictive settings functioned within the context of the law to improve women’s abortion experiences, rather than to save lives. They were implemented to support the formal healthcare system and were sometimes driven by religious ideology.^24^ Hotlines in these settings also had a more narrow scope of practice, focusing on the post-abortion period,^24^ as compared to hotlines in more restrictive settings.^14,25^

#### Theme 2: hotlines as an advocacy tool to mobilise activities and messaging

We found that hotlines engaging in advocacy activities and social networking facilitated greater reach of information to women. This helped to promote abortion-related discourse and shift it away from the narrative of clandestine abortions. Through the use of blogs, social media platforms such as Twitter and Facebook, and the creation of physical spaces where individuals could meet and network,^14,25^ community engagement was fostered to advocate for the decriminalisation of abortion. On average, hotlines received between 450 and 20,000 callers in one year.^14^ Since its creation in 2008, one hotline has had 2.5 million visitors to its website, where hotline staff and users could connect through the chat function.^14^ This hotline created more opportunities through various platforms to provide the opportunity for millions of people to engage and access information. In addition, it expanded to also create physical spaces for communities to organise and discuss advocacy strategies. Such forms of community organising provided an opportunity for hotlines to connect their users to abortion-friendly services, as well as to disseminate clinical abortion guidelines and protocols (including those related to abortion medication use) in local languages.

Political affiliation or messaging was particularly important to some hotlines. A few hotlines identified the political objective of promoting lesbianism as a form of rejection of patriarchy and heteronormativity.^14^ Through this narrative, these hotlines wanted to create a link between “pride in aborting” and “abortion lesbianizes” as a tool to fight stigma.^14^ Other hotlines’ identities were aligned with the socialist political party of their respective countries, with the goal of promoting decriminalisation of abortion and to make governments take responsibility for women’s health. For others, the goal was to remain outside the political sphere and focus solely on women’s autonomy, “not waiting for the State to grant women their ‘rights’”.^14,24,25^

An important feature of all the hotlines was balancing the visibility of their services with strategic messaging that was mindful of the context in which they were implemented.^14,24^ Whether this was in settings where abortion hotlines were part of community-based models outside the formal health system or those that were part of the formal care pathway, there was an underlying objective of normalisation of abortion in the public domain. In general, abortion hotlines were highlighted as powerful advocacy tools with the objective to improve care, destigmatise abortion and decrease maternal mortality and morbidity from unsafe abortion.

#### Theme 3: limitations of hotlines

For hotlines operating in a legally restrictive setting, their scope of practice was limited. Although hotlines in these settings provided harm reduction counselling and evidence-based information on the safe use of abortion medications, they were not able to provide women with the medications. Women utilising these hotlines frequently voiced concerns about abortion medications quality and possible scams^14,25^ as illustrated in the following quote: “misoprostol can be obtained on the black market for prices ranging from US $70–215, but as it is [an] under-the-table transaction, product quality is far from assured.”^25^

Community-based hotlines were also not able to track or influence what happened to women, including those whom they referred to the formal health system. Following referral, women sometimes reported experiencing mistreatment and stigma in healthcare facilities. Hotlines had no information as to how many women successfully completed their abortions. Some hotlines did try to evaluate their services, but it was difficult to know how many women were actually using the information or sharing it with others.^14^ In addition, community-based hotlines were limited in who they could reach and help; younger women who had access to the internet, women who were literate and those who lived in urban areas with reliable networks were more likely to utilise the hotlines.^14^ Hotlines were less likely to be utilised by women living in rural areas, migrant women speaking different dialects and older women.^14^

The US-based hotlines did not specifically address the challenges that women faced to access abortion; however, they attempted to address the myth that abortion carries longstanding negative emotional impacts.^24^ Though the hotlines provided episodic emotional support (acknowledging that women may experience initial difficulties following their abortion), they were limited in providing support to women with chronic mental health needs and with pre-existing conditions.^24^

Issues surrounding sustainability were something all hotlines had in common. Hotlines faced challenges to remain financially sustainable and to retain staff, and as a result their hours of operation were limited. For the most part, they received temporary financial support from international organisations and relied heavily on volunteers.^14,24,25^ The support tended to be temporary and hotlines considered alternatives to continue to operate. Through the various advocacy-related activities initiated by hotlines, additional resources were generated, including by charging for phone calls, selling tickets for raffles or accepting private donations.^14^

### Review of abortion hotlines

A total of 50 hotlines in the public domain were contacted; 16 abortion hotlines responded and completed the data extraction form. All except one of the hotlines that responded were also identified through the search of abortion hotlines available in the public domain. Countries represented were: Argentina, Canada, Indonesia, Kenya, Mexico, Nicaragua, Nigeria, Pakistan, Republic of Ireland, Uganda and the USA. Results received from the data extraction exercise are presented in Table 3. The majority of responding hotlines focus on providing information to individuals about abortion, using evidence-based guidance and some will give information about where abortion pills can be accessed. In addition to providing information about abortion, half of the hotlines (8/16; 50%) reported that they also provide comprehensive sexual health information, including information on contraception and sexually transmitted infections, and postpartum care. Other services provided by hotlines, not listed in Table 3, included funding for abortion travel (2/16; 13%), spiritual support (2/16; 13%), referral for legal support (1/16; 6%), and workshops (8/16; 50%).

**Table 3.** Data extraction results

Item^a^	Hotline responses	
	(n=16)	n (%)
***What does your hotline do?***		
Pre-abortion counselling Referrals to clinics that provide safe abortion Provide information about clinics that provide SA Send abortion pills to individual Inform where one can access abortion pills Provide information on how to use abortion pills Offer accompaniment at home or in-clinic Offer post-abortion counselling Offer referrals to clinics that provide post abortion care Provide information about clinics that provide post abortion care	12 13 13 6 13 13 8 11 10 12	75% 81% 81% 38% 81% 81% 50% 69% 63% 75%
***How do people reach you?***		
Phone Text Online Chat	16 11 9	100% 69% 56%
***Do you have a Standard Operating Procedures or best practices manual?***		
Yes No	13 2	81% 13%
***Does your hotline use clinical or other guidelines related to safe abortion care?***		
Yes If yes, which ones (n = 13)^b^ World Health Organization (WHO) National Abortion Federation (NAF) National Obstetrics & Gynecology Guidelines Marie Stopes International No	13 11 2 1 1 3	81% 85% 15% 8% 8% 19%
***Who is responsible for answering calls for your hotline?***		
Counsellors Nurses Pharmacists Midwives Non specialist doctors Specialist doctors Community health worker/ lay health workers Other (i.e. volunteers, feminist accompaniers/acompañantes, feminist activists)	8 1 0 1 1 0 3 7	53% 7% 7% 7% 20% 44%
***Does your hotline produce yearly reports?***		
Yes No	9 6	60% 40%

^a^ Some respondents provided multiple answers or did not answer at all and therefore the total number does not add to n = 16

^b^ Some listed more than one guideline

Narrative synthesis of the free-text responses received on the data extraction form is presented below.

#### Engagement with marginalised groups (i.e. youth, people with disabilities, migrant/refugee people)

Hotlines reported that their work expands into advocacy and community engagement with marginalised groups. To engage with people living with disabilities, one hotline uses educators who specialise in sexual education specifically for people with disabilities. To connect with youth, eight hotlines stated that they use social media (i.e. Facebook, WhatsApp groups) and conduct workshops either within the cities or at universities. They also engage youth through peer-to-peer counselling and will hire them to conduct dissemination activities directly. Four hotlines stated that they engage individuals through art and sports, including song, dance, use of radio and TV talk shows, and street graffiti activities. These hotlines engage indigenous and rural communities specifically, by ensuring hotline content is represented in various dialects and that information is disseminated widely, such as through radios. Delivering pamphlets, creating safe spaces for individuals to share their stories, and developing alliances with organisations where migrants may live were a few of the other activities listed by six hotlines.

#### Training of hotline staff

There was diversity in the ways that training was accomplished for hotline staff. All 16 hotlines highlighted the importance of continued professional development to maintain team morale and keep counsellors up to date. Some examples included peer-to-peer supervision, supervised calls, regular online training sessions and webinars, and opportunities for face-to-face trainings. One hotline specifically stated that their staff training lasts approximately six months, with 40 hours of theory, 40 hours of practice on the telephone, 40 hours of clinic support, and a visit to an abortion service provider. Three of the hotlines reported that they receive training support from reputable community-based, data-driven organisations. Five hotlines highlighted that their training is not restricted to abortion, but also includes other aspects of sexual and reproductive health and rights, STI prevention, contraception and legal frameworks supporting the sharing of information on abortion.

#### Self-description of hotline services

Representatives of hotlines were asked how they would describe their services. The most common words to describe hotline services included: self-care, self-management, feminist, accompaniment, de-medicalisation, non-medicalised and free. Of the 16 hotlines, 13 reported that their hotline specifically facilitates demedicalisation of abortion services and self-management of medical abortion with or without accompaniment. Furthermore, two were integrated as part of the formal care pathway and one was specifically identified as an abortion fund and practical support organisation.

## Discussion

This review highlights how context conditions the existence of hotlines; what their objectives are; how they operate; what is their scope of practice; and what issues exist in terms of their messaging and sustainability. Regardless of the legal context, hotlines’ unequivocal objective was to respond to a perceived unmet reproductive health need and thereby improve access to abortion care. Like other models of harm reduction implemented globally, our findings underline how hotlines operating parallel to the formal health system, do so in response to the legal context to facilitate safe self-managed abortions. They do so at the risk of legal repercussions and adapt their services to mitigate this risk of harm to themselves and to the women using their services. Our review sheds light on how hotlines operate in both liberal and restrictive legal settings. However, our findings also illustrate the scarcity of evidence relating to the impact of abortion hotlines in various contexts, as well as on hotline users’ and providers’ experiences.

Over the last decade, abortion hotlines have expanded as an innovative way to address legal and health systems’ shortcomings related to comprehensive abortion care.^27^ Similar to harm reduction programmes in Uruguay, Peru, Argentina and Nepal, which were developed to address high maternal mortality and morbidity rates due to unsafe methods, hotlines mitigate risks by providing abortion seekers with evidence-based counselling and information before and after their abortion.^9,8–30^ Our findings highlight existing knowledge that abortion hotlines in legally restrictive settings are an important example of a harm reduction model, with potential to decrease the negative effects of unsafe abortion and strengthen sexual and reproductive health and rights in the populations they serve, similar to other harm reduction models that have been studied, for example in Uruguay.^28^ Although we found that some hotlines struggle to reach marginalised populations, information received through the data extraction form demonstrates the efforts of hotlines to adapt their messaging and increase their reach to also include youth, migrants, sexual minorities and people with disabilities. This underscores their role as one tool that could facilitate a positive high-quality abortion experience. Moreover, community-based models, like those that include abortion hotlines, can facilitate person-centredness, dignity, autonomy, privacy, communication, support, compassionate care and trust.^31^ De-medicalising abortion provision in the context of community-based care requires continued work to destigmatise and decriminalise abortion care, so as to entrust women and ensure their reproductive rights are upheld. This not only requires access to evidence-based information and support, as highlighted by models like abortion hotlines, but advocacy at a global and national level to ensure access to affordable and quality assured medications and referral pathways to trusted facilities if and when a person may want or need it.

Though abortion hotlines in some settings are part of a harm reduction model of care, they also represent a low-fidelity, versatile innovation that can address access issues in challenging contexts, especially as they relate to information provision.^32^ For example, in humanitarian and fragile settings, and more recently during the pandemic, studies have identified that lessons learned by decades of implementation of innovations like hotlines should be adapted to the complex contexts of these settings.^32–34^ Our findings highlight that there may be a unique opportunity for these innovative models to transform traditional service delivery of safe abortion care, specifically including self-management alongside, rather than separate from, the formal health system.

Our findings suggest that sustainability is an issue for abortion hotlines which requires innovative solutions, although these challenges are not unique to abortion hotlines.^4,29^ Dependence on international funding agencies and varied health system financing structures (i.e. private versus public) are challenges faced by abortion hotlines. Based on our review, little data exists on this topic. By gathering more evidence about the value and impact of these models, governments and health systems may be more encouraged to invest in these models, distributing resources differently, and aligned with shared values and goals of person-centred, dignified and accessible healthcare delivery.^31^

Despite the breadth of information within this review, the review does have limitations. Included studies were few and diverse in their methodologies and outcomes. One key outcome was to elicit user experience; however, we found little data that highlighted this. Furthermore, the clandestine nature of abortion in legally restrictive settings also means that some hotlines operate in secrecy and we may therefore have missed many hotlines that exist worldwide. This may also be a reason why specific data on user experience has been difficult to obtain, as most users may prefer to stay anonymous and most may contact a hotline once, with no further follow-up. We may also have missed hotlines due to our limited ability to communicate in languages other than English, French and Spanish. Finally, the response rate to the data extraction form was less than 50%, although the hotlines that did respond were geographically diverse.

Further research is needed to understand the experiences of hotline providers and users, the role and impact of hotline messaging and advocacy, as well as studies that provide information on how abortion hotlines around the world influence abortion experiences and outcomes. Better understanding is needed of the values and preferences of people who seek abortions, and our findings highlight a major gap in this area, particularly when it comes to models of care that facilitate self-managed abortion. There are studies examining abortion seekers’ experiences utilising telemedicine models, community-based distribution of abortion medications, and accompaniment models, but there is limited research with a specific focus on abortion hotlines.^4,29,35–38^

## Conclusion

This review set out to describe abortion hotlines around the world, how they operate and how they facilitate access to safe abortion. Our findings show that the existence of abortion hotlines is conditioned by context, which influences everything from why they are implemented to how they operate, their messaging and what services they provide. While hotlines in legally restrictive settings generally provide a broad set of services, they are limited in their ability to ensure women access to quality medications, as well as their capacity to follow-up and evaluate their services. Our findings suggest that abortion hotlines play a large role in access to self-managed abortion, but further research is needed to understand both hotline user and provider experiences. In an era of increasing geopolitical tensions and global pandemics, our current systems require close examination and deliberate action to truly engage with women and communities to implement community-based models of care that can support and facilitate access to self-managed abortion care. Doing so has the potential to ensure that globally, no woman or girl will suffer from the consequences of unsafe abortions.

## Supplementary Material

Supplemental Data 1

Supplemental Data 2

Supplemental Data 3
