# Supplementary material for: Abortion hotlines around the world: a mixed-methods systematic and descriptive review
Source: Sex Reprod Health Matters. 2021 Apr 29;29(1):1907027. doi: 10.1080/26410397.2021.1907027 (PMC8097399; doi:10.1080/26410397.2021.1907027)
Supplement: Supplemental Data 1 [file ZRHM_A_1907027_SM2245.docx]

**Table 4.** Modified CASP Quality Assessment of Qualitative Studies

| **CASP Assessment Checklist** | | | |
| --- | --- | --- | --- |
| **Section A: Are the results valid?** | | | |
| **Study Author(s)** | **Casas L** | **Drovetta** | **Kimport** |
| 1. Was there a clear statement of the aims of the research? | **√** | **√** | **√** |
| 1. Is a qualitative methodology appropriate? | **√** | **√** | **√** |
| 1. Was the research design appropriate to address the aims of the research? | **\** | **√** | **√** |
| 1. Was the recruitment strategy appropriate to the aims of the research? | **√** | **\** | **√** |
| 1. Was the data collected in a way that addressed the research issue? | **\** | **\** | **√** |
| 1. Has the relationship between the researcher and participants been adequately considered? | **X** | **X** | **X** |
| **Section B: What are the results?** | | | |
| 1. Have ethical issues been taken into consideration? | **√** | **√** | **√** |
| 1. Was the data analysis sufficiently rigorous? | **\** | **\** | **√** |
| 1. Is there a clear statement of findings? | **X** | **X** | **√** |
| **Section C: Will the results help locally?** | | | |
| 1. How valuable is the research? | **√** | **√** | **√** |
| **Overall Quality Assessment** | Low | Low | Medium |

**√=**yes; **X**=No; **\**=Not specified or unclear
