# Supplementary material for: Abortion hotlines around the world: a mixed-methods systematic and descriptive review
Source: Sex Reprod Health Matters. 2021 Apr 29;29(1):1907027. doi: 10.1080/26410397.2021.1907027 (PMC8097399; doi:10.1080/26410397.2021.1907027)
Supplement: Supplemental Data 2 [file ZRHM_A_1907027_SM2241.docx]

**Database : PubMed**

**Database Provider : http://www.pubmed.gov**

**Database : Embase**

**Database Provider : http://www.embase.com**

**Database : CINAHL**

**Database Provider : http://www.ebsco.com**

**Database : PsycINFO / Ovid Emcare/ Maternity and Infant Care**

**Database Provider : http://www.ovid.com**

**Database : Global Index Medicus**

**Database Provider : http://www.globalhealthlibrary.net**

**Database : Google Scholar**

**Database Provider : <https://scholar.google.com/>**

**Database : Popline**

**Database Provider : https://www.popline.org/**

**Multi-disciplinary databases search**

**Database Provider :** [**http://www.ebsco.com**](http://www.ebsco.com)

| **#** | **PubMed** |
| --- | --- |
| **1** | "Hotlines"[Mesh] OR hotline [TW] OR hotlines [TW] OR helpline [TW] OR helplines [TW] OR "help lines" [TW] OR "help line" [TW] OR "telephone counseling" [TW] OR "telephone Counselling" OR "phone counselling" [TW] OR "phone counseling" [TW] |
| **2** | “Abortion, Induced"[Mesh] OR "induced abortion"[TW] OR "induced abortions"[TW] OR "abortion, missed"[MeSH Terms] OR "missed abortion"[TW] OR "missed abortions"[TW] OR "abortion, spontaneous"[MeSH Terms] OR "spontaneous abortion"[TW] OR "spontaneous abortions"[TW] OR "menstrual regulation"[TW] OR "abortion, legal"[MeSH Terms] OR "legal abortion"[TW] OR "legal abortions" [TW] OR "Postconception Fertility Control"[TW] OR Embryotomies[TW] OR Embryotomy[TW] OR "menstrual regulation"[TW] OR “abortion” [TW] OR "Pregnancy"[Mesh] OR “Pregnancy Complications"[Mesh]  OR “Sexually Transmitted Diseases"[Mesh]  OR "Abortion, Criminal"[Mesh] OR "Abortion, Induced"[Mesh] OR "Acquired Immunodeficiency Syndrome"[Mesh] OR HIV[Mesh] OR Postpartum period[Mesh] OR "Puerperal Disorders"[Mesh] OR "Obstetrics"[Mesh] OR "Parturition"[Mesh] OR "Puerperal"[TW] OR "Obstetrics"[TW] OR "Parturition"[TW] OR ”Abortion” [TW] OR ”Childbirth”[TW] OR ”Miscarriage” [TW] OR ”Pregnancy” [TW] OR ”Pregnancies” [TW] OR ”Postpartum” [TW] OR  ”Stillbirth” [TW] OR “sexually transmitted infection”[TW] OR “sexually transmitted”[TW] OR  "sexually acquired"[TW] OR ”HIV” [TW] OR "Acquired Immunodeficiency Syndrome"[TW] |
| **3** | Step 1 and Step 2 |
| **4** | \|  \|  \| \| --- \| --- \| |
