# Supplementary material for: Abortion hotlines around the world: a mixed-methods systematic and descriptive review
Source: Sex Reprod Health Matters. 2021 Apr 29;29(1):1907027. doi: 10.1080/26410397.2021.1907027 (PMC8097399; doi:10.1080/26410397.2021.1907027)
Supplement: Supplemental Data 3 [file ZRHM_A_1907027_SM2238.docx]

**Annex 1**

**Hotline Questionnaire – English**

1. *What does your hotline do?* (please check all boxes that apply)

[ ] We offer accompaniment at home or in-clinic

[ ] We offer post abortion counselling

[ ] We offer referrals to clinics that provide post abortion care

[ ] We provide information about clinics that provide post abortion care

[ ] Other (please specify) _______________________________

[ ] We offer pre-abortion counselling

[ ] We offer referrals to clinics that provide safe abortion

[ ] We provide information about clinics that provide safe abortion

[ ] We send abortion pills to the individual

[ ] We inform where one can access abortion pills

[ ] We provide information on how to use abortion pills

*B.* *How do people reach you?* (please check all boxes that apply)

[ ] Calls

[ ] Text

[ ] Online chat

[ ] Other (please specify)

*C. Do you have standard operating procedures (SOP) or a best practices manual?*

[ ] Yes If Yes, what does your SOP or best practices manual cover in general

[ ] No

*D. Does your hotline use clinical or other guidelines related to safe abortion care?*

[ ] Yes If Yes, which ones?

[ ] No

*E. What strategies does your hotline use to assist and/or engage: youth (10 – 24), people with disabilities, migrant/refugee people, other hard to reach groups?*

*F. Who is responsible for answering calls for your hotline?*

[ ] Non Specialist Doctors

[ ] Specialist Doctors

[ ] Community Health Worker/ Lay Health Workers

[ ] Other (please specify): ____________________

[ ] Counselors

[ ] Nurses

[ ] Pharmacists

[ ] Midwives

*G. Could you briefly describe how your hotline staff are trained?*

*H. How would you self-describe your service i.e. integrated with medical system, demedicalized care, self-management, etc.?*

*I. Does your hotline produce yearly reports?*

[ ] Yes If you are you willing to share, please attach the document in your email to us

[ ] No

**Hotline Questionnaire – Spanish**

*A. Qué servicios ofrece su línea telefónica?* (por favor, marque todo lo que aplique)

[ ] Ofrecemos acompañamiento en el hogar o en la clínica

[ ] Ofrecemos consejería posaborto

[ ] Ofrecemos derivaciones a clínicas que ofrecen servicios posaborto

[ ] Ofrecemos información sobre clínicas que proveen servicios posaborto

[ ] Otro (por favor, especifique) _______________________________

[ ] Ofrecemos consejería preaborto

[ ] Ofrecemos derivaciones a clínicas que proveen abortos seguros

[ ] Ofrecemos información sobre clínicas que proveen abortos seguros

[ ] Enviamos tratamiendo médico del aborto a las personas

[ ] Ofrecemos información sobre dónde se pueden obtener las pastillas para el tratamiento médico del aborto

[ ] Ofrecemos información sobre cómo usar las pastillas para el tratamiento médico del aborto

*B.* *¿Cómo hace la gente para contactarlos?* (por favor, marque todo lo que aplique)

[ ] Llamadas telefónicas

[ ] Mensajes de texto

[ ] Servicios de mensajería en línea

[ ] Otro (por favor, especifique)

*C. ¿Tienen un manual de operaciones o mejores prácticas?*

[ ] Sí Si SÍ, en general, ¿qué cubre su manual de operaciones o de buenas practices

[ ] No

*D. ¿Utilizan directrices clínicas u otras para informar su línea telefónica en cuanto al cuidado de aborto seguro?*

[ ] Sí Si SÍ, ¿cuáles?

[ ] No

*E. ¿Qué estrategias utiliza su línea telefónica para asistir y/o atraer a: jóvenes (10 – 24 años), personas con discapacidad, migrantes/refugiados, otros grupos de personas difíciles de acceder?*

*F. Quien es responsable en responder a las llamadas en su línea telefónica?*

[ ] Consejeros/as

[ ] Enfermero/as

[ ] Farmacéuticos/as

[ ] Parteras/Obstétricas

[ ] Médicos generalistas

[ ] Médicos especialistas

[ ] Trabajadores comunitarios/Trabajadores legos de salud

[ ] Otro (por favor, expecifique): ____________________

*G. Por favor describa cómo es capacitado el personal de su línea telefónica.*

*H. ¿Cómo describiría su servicio, por ejemplo: sistema médico integrado, cuidado no medicalizado, autocuidado, etc.?*

*I. ¿Realizan reportes anuales sobre su línea telefónica?*

[ ] Sí Si están dispuestos a compartirlo, por favor adjúntenlo en su correo electrónico

[ ] No

**Hotline Questionnaire - French**

A. *Que fait votre Ligne Verte (veuillez cocher toutes les cases qui s'appliquent) ?*

[ ] Nous offrons un accompagnement à domicile ou en clinique.

[ ] Nous offrons du counselling post-avortement

[ ] Nous offrons des références vers des cliniques qui offrent des services de soins post-avortement.

[ ] Nous fournissons de l'information sur les cliniques qui offrent des services de soins post-avortement.

[ ] Autre (veuillez préciser) _______________________

[ ] Nous offrons des counselling pré-avortement

[ ] Nous offrons des références vers des cliniques qui offrent des services d'avortement sécurisé.

[ ] Nous fournissons des informations sur les cliniques qui pratiquent l'avortement sécurisé.

[ ] Nous envoyons des médicaments (pilules) pour l'avortement à la personne concernée.

[ ] Nous informons où l'on peut avoir accès aux médicaments d'avortement (pilules).

[ ] Nous fournissons de l'information sur la façon d'utiliser les médicaments d'avortement (pilules).

Traduit avec www.DeepL.com/Translator

*B.* *Comment les gens vous rejoignent-ils ? (veuillez cocher toutes les cases qui s'appliquent)*

[ ] coups de téléphone

[ ] Texto / SMS

[ ] Chat en ligne

[ ] Autre (veuillez préciser)

*C. Avez-vous des procédures d'utilisation normalisées (PON) ou un manuel des meilleures pratiques ?*

[ ] Oui Si oui, que couvre votre PON ou votre manuel des meilleures pratiques en général ?

[ ] Non

*D. Votre Ligne Verte utilise-t-elle des directives cliniques ou d'autres directives relatives aux soins d'avortement sécurisés ?*

[ ] Oui Si oui, lesquelles?

[ ] Non

*E. Quelles stratégies votre Ligne Verte utilise-t-elle pour aider et/ou mobiliser : les jeunes (10-24 ans), les personnes handicapées, les migrants/réfugiés, les autres groupes difficiles à atteindre ?*

*F. Qui est responsable de répondre aux appels de votre Ligne Verte ?*

[ ] Médecins non spécialistes

[ ] Médecins spécialistes

[ ] Agents de santé communautaire/ Volontaire communautaire, etc.

[ ] Autre (veuillez préciser): ____________________

[ ] conseillers

[ ] infirmiéres

[ ] Pharmaciens

[ ] Sage-femmes

*G. Pouvez-vous décrire brièvement comment votre personnels de Ligne Verte sont formés?*

*H. Comment décririez-vous votre service, c.-à-d. intégré au système médical, aux soins démédicalisés, à l'autogestion, etc.*

*I. Votre Ligne verte produit-elle des rapports annuels?*

[ ] Oui Si vous êtes prêt à partager le rapport, veuillez le joindre à votre courriel

[ ] Non
